# A systematic review and meta-analysis of the prevalence and risk factors of oral frailty among older adults

**DOI:** 10.1186/s12903-026-08435-y

**Published:** 2026-05-02

**Authors:** Meng Li, Yahui Wang, Lingguo Lei, Wanqian Zhao, Shaoling Xiao

**Affiliations:** 1 School of Nursing, Changzhi Medical College, Changzhi, China; 2 Pharmacy Intravenous Admixture Services, Heping Hospital Affiliated to Changzhi Medical College, Changzhi, China; 3 Department of Science and Education, Heping Hospital Affiliated to Changzhi Medical College, No. 161, Jiefang East Street, Luzhou District, Changzhi City, Shanxi Province 046000 China

**Keywords:** Older adults, Oral frailty, Prevalence, Risk factors, Meta-analysis

## Abstract

**Objectives:**

To systematically review the prevalence of oral frailty among older adults and its associated risk factors.

**Method:**

This systematic review and meta-analysis was prospectively registered with PROSPERO (CRD420261288065) and conducted in accordance with PRISMA guidelines. We developed a search strategy and systematically searched PubMed, Embase, Web of Science, MEDLINE, the Cochrane Library, and CINAHL from inception to August 1, 2025. Studies investigating the prevalence of oral frailty or its risk factors among older adults were included. Two reviewers independently performed study screening, data extraction, and quality assessment, and data were analyzed using RevMan 5.4 and Stata 14.0. This study was registered in PROSPERO.

**Results:**

A total of 16 studies involving 13,030 participants were included. The pooled prevalence of oral frailty was 32% (95% CI: 25%–41%), with substantial heterogeneity (*I*² = 98.85%). Subgroup analyses showed that prevalence varied by country, study design, assessment instrument, and publication year (all *p* < 0.05). Univariable meta-regression indicated that the assessment instrument was the primary source of heterogeneity (R² = 56.1%, *p* < 0.001). Pooled analyses of risk factors showed that physical frailty (95% CI: 1.41–4.62), age (95% CI: 1.08–1.11), having fewer than 20 remaining teeth (95% CI: 2.27–21.00) and female sex (95% CI: 1.22–1.91) were associated with an increased risk of oral frailty (all *p* < 0.05).

**Conclusions:**

Oral frailty is prevalent among older adults and is associated with demographic characteristics, overall frailty status, and underlying oral structural conditions. Clinical and community settings should focus on older adults at high risk of oral frailty, particularly those with fewer than 20 remaining teeth, physical frailty, advanced age, or female sex. Early identification should be prioritized, and multidimensional interventions—including oral function and dentition assessment, nutritional support, frailty management, and targeted oral health education—should be implemented through multidisciplinary collaboration to reduce risk and improve quality of life.

**Supplementary Information:**

The online version contains supplementary material available at 10.1186/s12903-026-08435-y.

## Background

Global population aging continues to accelerate. In 2022, people aged ≥ 65 years accounted for 9.7% of the world’s population, and this proportion is projected to increase to 16.4% by 2050 [1]. Against this backdrop, the health burden associated with frailty and functional decline among older adults is becoming increasingly pronounced. Oral health is closely associated with nutritional status, quality of life, and social functioning [2–4]. However, with advancing age, conditions such as tooth loss, dental caries, and periodontal disease become more common, leading to progressive deterioration of oral structure and function [5, 6]. As a comprehensive phenotype reflecting multidimensional declines in oral function among older adults, oral frailty has become a priority that warrants urgent attention in research and practice related to healthy aging [7].

Oral frailty refers to a series of degenerative processes in oral functions that occur with advancing age, including reduced tooth quantity, decline in oral hygiene and function, decreased attention to oral health, weakened physical and mental reserve capacity, and dysphagia [8–10]. Oral frailty often triggers a chain of reactions, such as reduced nutrient intake, weight loss, physical weakness, and cognitive decline, which further lead to a range of adverse health outcomes, including physical frailty, functional disability, increased hospitalization and fall risk, reduced quality of life, and even higher mortality [11–13]. Notably, existing studies suggest that oral function indicators associated with oral frailty exhibit certain interventability [14]. Timely identification of high-risk factors can facilitate the development of targeted intervention strategies to mitigate their impact.

In recent years, multiple systematic reviews and meta-analyses have integrated evidence on the prevalence and risk factors of oral frailty in the elderly. However, these studies exhibit several methodological limitations. Substantial variations in assessment instruments and operational definitions of oral frailty across studies may have amplified between-study heterogeneity [15]. Some reviews employed earlier search cutoffs and may therefore have missed high-quality studies published subsequently [16]. In addition, some meta-analyses included a large number of Chinese-language studies, which may limit the generalizability of their conclusions [17]. Therefore, this study aims to conduct a systematic review and meta-analysis, incorporating studies published in English that evaluate oral frailty using Oral Frailty Index-6 (OFI-6) or Oral Frailty Index-8 (OFI-8), combining their prevalence rates, and systematically summarizing the relevant risk factors, while exploring sources of heterogeneity through subgroup analyses and univariable meta-regression. This work is expected to provide evidence to inform surveillance, risk assessment, and the development of prevention and control strategies for oral frailty among older adults.

## Methods

This meta-analysis adhered to the PRISMA guidelines [18]. This systematic review was prospectively registered with PROSPERO, an international prospective register of systematic reviews, under registration number CRD420261288065, the registration record is available at https://www.crd.york.ac.uk/prospero/.

### Search strategy

This study systematically searched PubMed, Embase, Web of Science, MEDLINE, Cochrane Library, and CINAHL, with the search time range covering the databases from their establishment to August 1, 2025. The search strategy combined MeSH terms with free-text keywords and was tailored appropriately for each database. The terms used included: “aged”, “elderly”, “older adult*”, “oral frailty”, “impact*”, “cause*”, and “reason*”. Manual searches and reference checks were also performed to supplement the database searches. Only English-language studies were included, with no restrictions on publication status. Grey literature was not systematically searched. For the complete search strategy, please refer to Supplementary Table 1.

### Inclusion and exclusion criteria

The inclusion criteria for studies in this systematic review were as follows: (1) participants were older adults aged ≥ 60 years; (2) oral frailty was assessed using a clearly defined scale or instrument, such as the Oral Frailty Index-6 (OFI-6, a professional assessment requiring objective measurement of oral function) or Oral Frailty Index-8 (OFI-8, a self-reported questionnaire covering oral function, behaviors, and social participation); (3) the study reported the prevalence and/or risk factors of oral frailty; (4) observational studies (cross-sectional or cohort) published in English. This study included only English-language publications using the OFI-6/OFI-8 instruments, to enhance methodological homogeneity and ensure comparability across studies.

The exclusion criteria were as follows: (1) non-original studies such as reviews, conference abstracts, and case reports; (2) studies for which the full text was unavailable; (3) studies with populations that did not meet the inclusion criteria; (4) studies with assessment tools or outcome measures that were not aligned with the review objectives. When information extraction was incomplete, we attempted to contact the original authors to obtain supplementary data, studies for which the authors could not be reached were excluded.

### Study selection and data extraction

Study selection and data extraction were conducted independently by two reviewers, and all retrieved records were imported into EndNote X9 for duplicate checking. The reviewers screened studies according to predefined inclusion and exclusion criteria, and any discrepancies were resolved through discussion with a third reviewer to reach consensus.

Using a standardized data-extraction form, the two reviewers independently extracted data and cross-checked the results to ensure accuracy. Extracted information included first author, year of publication, country, study design, sample size, mean age, study setting, assessment instrument, prevalence of oral frailty, and associated risk factors.

### Quality assessment

Two researchers independently evaluated the methodological quality. For cohort studies, the Newcastle-Ottawa Scale (NOS) was used, which evaluates study quality across three domains: selection, comparability, and outcome assessment [19]. The quality grades are classified as follows: 7–9 points indicate high quality, 5–6 points indicate medium quality, and 0–4 points indicate low quality. For cross-sectional studies, the Agency for Healthcare Research and Quality (AHRQ) criteria was applied. This 11-item scale evaluates aspects including study information sources, inclusion/exclusion criteria, study duration and continuity, blinding of personnel, quality assurance assessment, confounding factors and missing data, response rate, and patient completeness. The quality grade is divided into: 8–11 points for high quality, 4–7 points for medium quality, and 0–3 points for low quality [20].

### Statistical analysis

Statistical analyses were performed using RevMan 5.4 and Stata 14.0. The pooled prevalence of oral frailty was expressed as a proportion with its 95% confidence interval (CI). For risk factors eligible for meta-analysis, we prioritized the most comprehensively adjusted models, and only the corresponding adjusted odds ratios (ORs) with 95% CIs were extracted; unadjusted estimates were not included in the pooled analysis, to account for potential confounding. Between-study heterogeneity was assessed using Cochran’s *Q* test and the *I*² statistic (with *p* < 0.10 in the *Q* test considered indicative of heterogeneity). A fixed-effects model was applied when *I*² ≤ 50%, whereas a random-effects model was used when *I*² > 50% to pool the study results. Sources of heterogeneity were explored through subgroup analyses and univariable meta-regression. Sensitivity analyses were conducted for the primary outcomes by sequentially omitting one study at a time. When an outcome included at least 10 studies, publication bias was assessed using funnel plots and Egger’s test, with *p* < 0.05 indicating the presence of small-study effects. All tests were two-sided, and *p* < 0.05 was considered statistically significant.

## Results

### Study selection

**Fig. 1:**
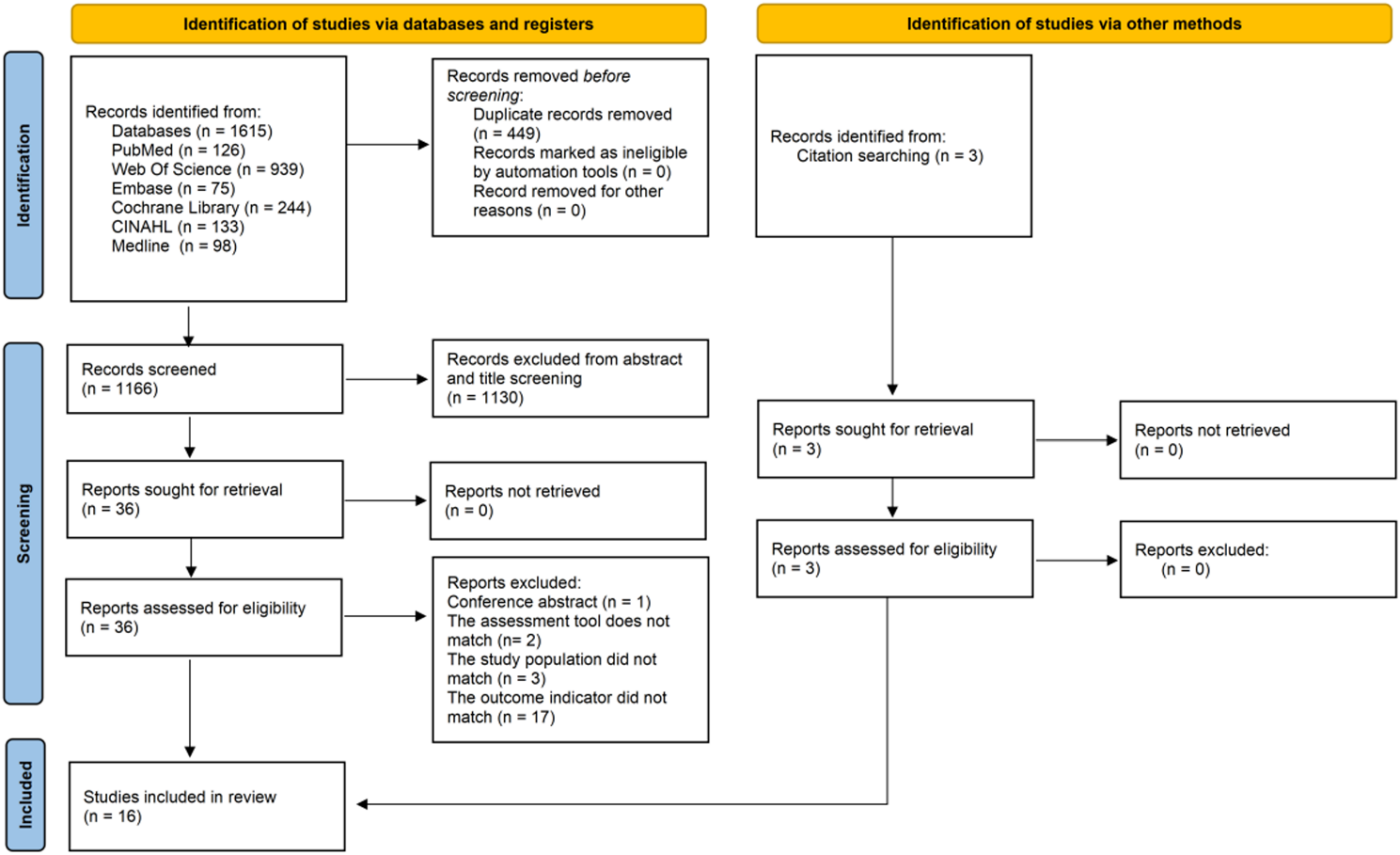
PRISMA flow diagram showing the study selection process

A total of 1,615 records were identified through a systematic database search. After removing duplicates, 449 records were excluded, and 1,166 records underwent initial screening. Based on the predefined inclusion and exclusion criteria, 1,130 records were excluded after title and abstract review. Consequently, 36 articles were preliminarily selected for full-text review and eligibility assessment. Of these, 1 conference abstract, 2 studies with unclear assessment tools (not OFI-6/8), 3 studies with mismatched populations, and 17 studies with nonconforming outcomes were excluded (Supplementary Table 2). Three additional studies were identified through citation tracking. Full texts for all studies entering full-text evaluation—including the 3 newly identified studies—were successfully obtained, and all eligibility criteria were verified. Ultimately, 16 studies [4, 10, 13, 21–33] were included in the final analysis. The detailed literature screening process is illustrated in Fig. 1.

### Characteristics and quality assessment of the included studies

The characteristics of the 16 studies included in this review are summarized in Table 1, of which 14 were cross-sectional studies and 2 were cohort studies. The publication time of the research was concentrated between 2020 and 2025, including 10 studies from Japan, 5 studies from China, and 1 study from India. All participants were older adults, and the primary recruitment sources for the included studies were community settings, hospitals, or outpatient clinics. The sample sizes varied significantly, ranging from 111 to 3,222 cases. Regarding the tools for assessing oral frailty, 8 studies used the OFI-8 scale and 8 studies used the OFI-6 scale. Except for 1 study (Kumar [26]), all other studies reported the prevalence of oral frailty. The Kumar [26] study did not report overall prevalence and was therefore excluded from the pooled prevalence analysis, but it was retained for risk factor analysis.

**Table 1:** Characteristics of included studies

First author year	Country/Setting	Study type	Mean age	Sample size	Measurement	Prevalence (%)	Risk factor	Quality score
Kumar 2023 [26]	India/Community	cross-sectional	66.72 ± 6.86	310	OFI-8	ND	1 2	6
Luo 2025 [10]	China Nanchong/hospitals	cross-sectional	71.44 ± 7.47	431	OFI-8	32.95	1 3 4 5 6 7	8
Tian 2025 [33]	China Taiyuan/hospitals	cross-sectional	≥ 60	464	OFI-8	45.9	1 8 9 10 11 12 13	5
Hironaka 2020 [21]	Japan Tokyo/Community	cross-sectional	73.3 ± 6.6	682	OFI-6	9.5	1 14 15 16 17 18	8
Komatsu 2021 [13]	Japan/Community	cross-sectional	72.8 ± 5.5	380	OFI-6	14	12 19	7
Ohara 2020 [22]	Japan Tokyo/Community	cross-sectional	79.1 ± 4.5	722	OFI-6	19.3	20	8
Nishimoto 2023 [27]	Japan/Community	Longitudinal study	72.2 ± 5.1	1234	OFI-6	23.1	21	8
Hoshino 2021 [23]	Japan/Community	cross-sectional	75.9 ± 6.3	481	OFI-6	21.2	22	8
Nakagawa 2024 [28]	Japan/Clinic	cross-sectional	79.9 ± 4.3	2727	OFI-6	44.3	1 12 18 22 23 24 39	7
Iwasaki 2020 [4]	Japan Tokyo/Community	longitudinal study	76.4 ± 4.1	466	OFI-6	14.4	25	8
Yamamoto 2022 [25]	Japan/dental clinics	cross-sectional	≥ 65	595	OFI-6	23.9	1 6 26 27	6
Tamaki 2024 [29]	Japan/Community	cross-sectional	80	3222	OFI-8	37.8	6 28 29 30 31	7
Liu 2025 [32]	China/hospitals	cross-sectional	≥ 60	180	OFI-8	39.4	22	5
Wang 2024 [30]	China Yanji/Community	cross-sectional	60–92	478	OFI-8	71.6	1 12 24 32 34 35 36 37 38 40	6
Zhang 2025 [31]	China/Community	cross-sectional	≥ 60	547	OFI-8	52.8	6 7 12 33	5
Ishii 2022 [24]	Japan/hospitals	cross-sectional	75–92	111	OFI-8	53.2	10	6

ND, not described. Risk factor: 1 is age; 2 is prosthetic status of the upper teeth; 3 is glycated hemoglobin (HbA1c ≥ 7%); 4 is dysphagia; 5 is poor oral health; 6 is remaining teeth (< 20 or ≥ 20); 7 is oral health-related self-efficacy (GSEOH); 8 is smoking; 9 is physical activity; 10 is duration of diabetes mellitus; 11 is polypharmacy; 12 is physical frailty; 13 is oral health score; 14 is mini-nutritional assessment short form (MNA-SF); 15 is stroke; 16 is social frailty; 17 is prefrailty; 18 is number of medications used; 19 is gait speed; 20 is eating alone; 21 is severe periodontitis; 22 is dietary variety; 23 is simplified nutritional appetite questionnaire; 24 is female; 25 is deteriorating nutritional status; 26 is difficulty eating tough foods; 27 is choking; 28 is poor dental plaque; 29 is oral malodor; 30 is family dental clinic; 31 is oral concern; 32 is ethnicity; 33 is family income; 34 is number of chronic diseases; 35 is body mass index; 36 is drinking; 37 is sleep disorders; 38 is attitudes towards aging; 39 is dietary diversity score; 40 is personal monthly income

Assessment using the AHRQ tool indicated that the scores of 14 cross-sectional studies ranged from 5 to 8, with an average of 6.57, 4 studies met the high-quality standard (≥ 8), while the remainder were of moderate quality (Supplementary Table 3 A). The two cohort studies assessed using the NOS scale each scored 8, classifying them as high-quality studies (Supplementary Table 3B).

### Prevalence of oral frailty

**Fig. 2:**
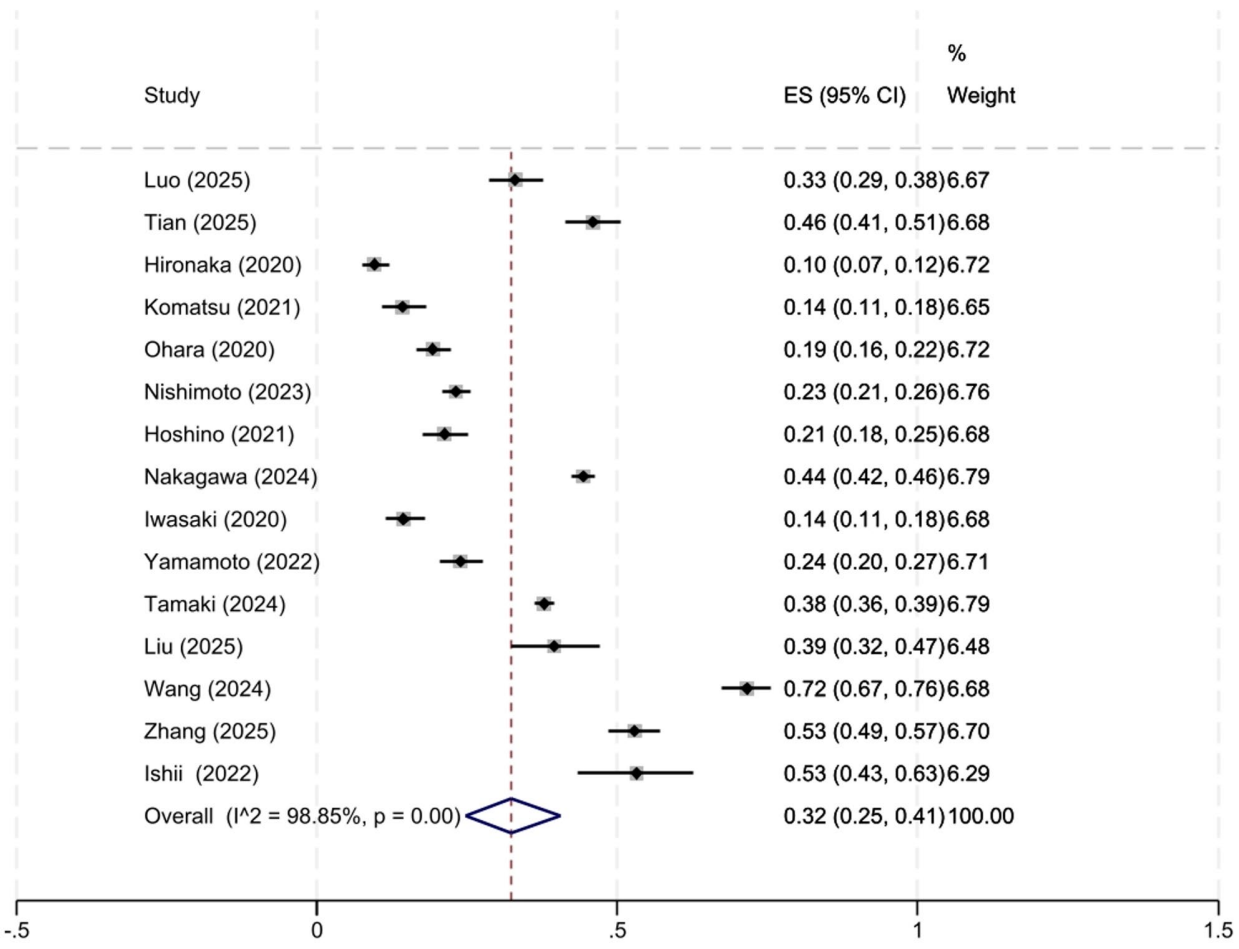
Forest plot of prevalence of oral frailty among older adults

A total of 15 studies reported the prevalence of oral frailty, with substantial variation across individual studies (9.5%–71.6%). Given the high heterogeneity (*I*² = 98.85%, *p* < 0.05), a random-effects model was used for pooling. The pooled prevalence was 32% (95% CI: 25%–41%) (Fig. 2). Due to high differences across studies, the pooled prevalence should be considered only as a general descriptive estimate, rather than an exact measure applicable to all populations and settings.

### Risk factors for oral frailty (meta-analyzable factors)

#### Physical frailty

**Fig. 3:**
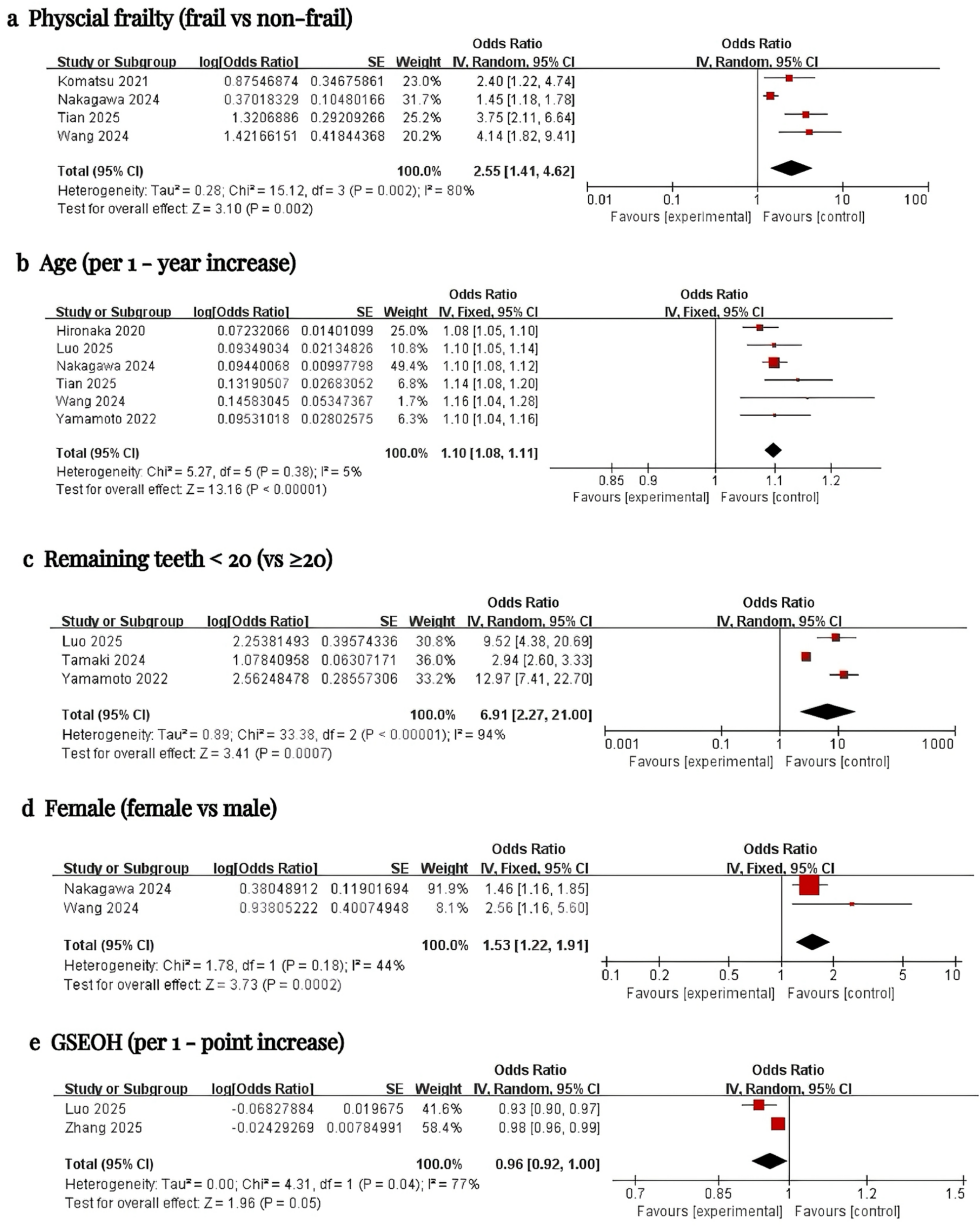
Forest plot results of oral frailty risk factors: **a** Physical frailty, **b** Age, **c** Remaining teeth <20, **d** Female, **e** GSEOH

Four studies were included in the meta-analysis; substantial heterogeneity was observed (*I*² = 80%), and a random-effects model was applied. The pooled results showed a significant positive association between physical frailty and oral frailty (OR = 2.55, 95% CI: 1.41–4.62, *p* = 0.002, Fig. 3a).

#### Age

Six studies were included in the meta-analysis; heterogeneity was negligible (*I*² = 5%), and a fixed-effects model was used. The pooled results indicated that each 1-year increase in age was associated with an increased risk of oral frailty (OR = 1.10, 95% CI: 1.08–1.11, *p* < 0.001, Fig. 3b).

#### Fewer than 20 remaining teeth

Three studies were included in the meta-analysis; substantial heterogeneity was present (*I*² = 94%), and a random-effects model was applied. Compared with individuals with ≥ 20 remaining teeth, older adults with < 20 remaining teeth had a significantly higher risk of oral frailty (OR = 6.91, 95% CI: 2.27–21.00, *p* = 0.0007, Fig. 3c).

#### Female

Two studies were included in the meta-analysis; heterogeneity was not substantial (*I*² = 44%), and a fixed-effects model was used for pooling. The pooled results showed that female sex was significantly positively associated with oral frailty (OR = 1.53, 95% CI: 1.22–1.91, *p* = 0.0002, Fig. 3d).

#### Geriatric self-efficacy for oral health (GSEOH)

Two studies were included in the meta-analysis; substantial heterogeneity was observed (*I*² = 77%), and a random-effects model was applied. The pooled association showed borderline statistical significance (OR = 0.96, 95% CI: 0.92–1.00, *p* = 0.05, Fig. 3e).

### Other risk factors (descriptive synthesis)

In addition, 35 other risk factors were identified from single-study evidence, spanning multiple domains, as detailed below: (1) demographic sociological factors: family income, personal monthly income, ethnicity, social frailty; (2) oral health status: prosthetic status of the upper teeth, decayed teeth, oral malodor, family dental clinic, oral concerns, difficulty eating tough foods, dysphagia, poor oral health, oral health score, severe periodontitis, poor dental plaque; (3) health status and physical function: prefrailty, number of chronic diseases, body mass index, the aging attitude, stroke, number of medications, polypharmacy, sleep disorders, choking, duration of diabetes mellitus, gait speed; (4) nutritional status and lifestyles: mini nutritional assessment-short form (MNA-SF) scores, deteriorating nutritional status, simplified nutritional appetite questionnaire, glycated hemoglobin (HbA1c) ≥ 7%, drinking, eating alone, physical activity, smoking, dietary variety.

### Sensitivity analysis and publication bias

Sensitivity analyses were conducted using a leave-one-out approach for the pooled prevalence of oral frailty and for pooled risk factors synthesized from at least three studies. For the prevalence analysis, after sequentially removing any single study, the recalculated pooled prevalence remained close to the overall estimate, with highly overlapping confidence intervals, and no individual study was found to materially influence the overall result, indicating good robustness of the pooled prevalence estimate (Supplementary Fig. 1). For the risk-factor analyses, sensitivity analyses of physical frailty, age, and having fewer than 20 remaining teeth showed that sequential omission of individual studies did not materially change the direction or statistical significance of the pooled effect sizes (Supplementary Fig. 2). Although some fluctuation in effect estimates was observed for certain factors due to the relatively small number of included studies, the overall association patterns remained consistent, suggesting methodological stability of the pooled results for the main risk factors.

Publication bias was assessed for prevalence estimates that included 10 or more studies, and the funnel plot showed no marked asymmetry (Supplementary Fig. 3). Egger’s test indicated no significant publication bias (*t* = − 0.64, *p* = 0.533).

### Subgroup analysis and univariate meta-regression

To explore sources of heterogeneity in the pooled prevalence of oral frailty, subgroup analyses were conducted according to country, mean age, study design, assessment instrument, publication year, sample size, and study setting (Supplementary Table 4). In addition, meta-regression was performed to examine the effects of these covariates on between-study variance. (Supplementary Table 5).

Subgroup analyses showed that the pooled prevalence in studies from China was 48.7% (95% CI: 0.352–0.623), markedly higher than that in studies from Japan 24.9% (95% CI: 0.173–0.334). The pooled prevalence in cross-sectional studies was 34.7% (95% CI: 0.263–0.437), which was substantially higher than that in cohort studies 20.5% (95% CI: 0.187–0.225). The prevalence estimated using the OFI-8 47.6% (95% CI: 0.376–0.578) was significantly higher than that assessed with the OFI-6 20.6% (95% CI: 0.121–0.306). Studies published during 2023–2025 reported a significantly higher prevalence 43.3% (95% CI: 0.348–0.520) than those published in 2022 or earlier 20.6% (95% CI: 0.146–0.274). No statistically significant differences in prevalence were observed across subgroups defined by mean age, sample size, or study setting (all *p* > 0.05). Univariable meta-regression indicated significant associations of country, assessment instrument, and publication year with the pooled prevalence, with the assessment instrument explaining the largest proportion of between-study heterogeneity (R² = 56%).

## Discussion

This systematic review and meta-analysis included 16 studies (*n* = 13,030), of which 15 reported the prevalence of oral frailty, ranging from 9.5% to 71.6%. Using a random-effects model, the pooled prevalence of oral frailty among older adults was 32% (95% CI: 25%–41%), indicating that oral functional decline has become an important issue affecting both oral and overall health in older populations.

Notably, most included studies were cross-sectional and of moderate quality, potentially affected by incomplete data, inadequate control of confounding factors, or sample selection bias, which could influence estimates of prevalence and risk factors. This study included only English-language publications, which may introduce language bias, high-quality non-English studies were not included, potentially affecting the comprehensiveness of the findings and their cross-cultural generalizability.

Although substantial between-study heterogeneity was observed, sensitivity analyses indicated that the pooled results were not unduly influenced by any single study, supporting the robustness of our overall findings.

To explore sources of heterogeneity, we examined study-level characteristics and found that country, study design, assessment instrument, and publication year were associated with prevalence estimates. Univariable meta-regression showed that country, assessment instrument, and publication year remained significant, with the assessment instrument explaining the largest proportion of between-study variability (R² = 56%). Nevertheless, substantial heterogeneity remained in the study results, suggesting that additional unmeasured factors—such as sampling strategies, environmental or cultural contexts, and variations in instrument implementation—may also have contributed. Therefore, the pooled prevalence should be considered a descriptive estimate, and interpretations should be made with caution.

With respect to assessment instruments, our findings are consistent with previous evidence, studies using the OFI-8 reported a higher pooled prevalence of oral frailty than those using the OFI-6 [34]. Notably, among the included studies, the OFI-8 self-reported questionnaire was more frequently used in Chinese studies, whereas the OFI-6 professional assessment tool predominated in Japanese studies. The OFI-8 comprises eight self-reported items covering domains such as functional oral health, oral health–related behaviors, and social participation, which may increase sensitivity for identifying individuals with mild or early functional decline [35]. The OFI-6 consists of six items, and its assessment must be administered by oral health professionals and requires appropriate dental equipment to ensure the accuracy and reliability of the procedures. It focuses more specifically on tooth count, oral articulation, tongue pressure, and masticatory and swallowing function [36]. Differences in the choice of assessment tools may partially explain the discrepancies in prevalence estimates between Chinese and Japanese studies and contribute to increased overall heterogeneity, therefore, caution is warranted when interpreting cross-national comparisons.

The overall prevalence of oral frailty among older adults was higher in China than in Japan. This difference may be driven by multiple factors and may partly reflect methodological differences, including the use of different assessment instruments and variations in sampling approaches. In addition to these methodological factors, contextual factors may also play a role. For example, Japan provides dental services through universal health insurance and has extended services for older adults to include home-based oral care [37], whereas China may differ substantially in the coverage and accessibility of preventive dental services [38].

Study design may also influence prevalence estimates. Cross-sectional studies capture prevalence at a specific time point, whereas longitudinal studies can observe the onset and progression of oral frailty over follow-up [39, 40], differences in temporal perspective and sample selection between these designs may lead to inconsistencies in prevalence levels and their variability. However, given the limited number of cohort studies, this difference requires confirmation with additional longitudinal evidence.

Stratified analyses by publication year showed that studies published during 2023–2025 reported a higher pooled prevalence than those published before 2022. This temporal pattern may partly reflect methodological changes, including the broader adoption of screening instruments that tend to yield higher prevalence estimates and the evolution of assessment practices [35].

After pooling repeatedly reported risk factors, we found that age was positively associated with oral frailty, with the risk of oral frailty increasing significantly as age increased, consistent with previous systematic reviews that have identified advanced age as a key risk factor for oral frailty [15]. This difference may be partly explained by age-related degenerative changes in oral structures and function in older adults, along with declines in sensorimotor control involved in mastication and swallowing. In addition, reduced manual dexterity in older individuals may compromise their ability to remove dental plaque during daily oral hygiene practices, making it difficult to maintain good oral hygiene and thereby undermining the stability of oral function [41].

Physical frailty is an important risk factor for oral frailty, consistent with the findings of Zhu et al. [42]. These two conditions may exert bidirectional effects through shared pathophysiological mechanisms. Physical frailty is often accompanied by sarcopenia, mobility limitations, fatigue, and an increased risk of malnutrition, which may not only compromise the ability to perform routine oral care but also impair the muscle groups involved in mastication and swallowing [43, 44]. Conversely, impaired oral function can restrict food choices, reduce dietary diversity, exacerbate malnutrition, and further aggravate physical frailty, thereby creating a mutually reinforcing vicious cycle [45].

Having fewer than 20 remaining teeth was also significantly associated with oral frailty, consistent with previous findings [46], suggesting that dental arch integrity is an important structural basis for maintaining normal oral function. Insufficient tooth number may reduce masticatory efficiency, leading older adults to prefer softer-textured foods and thereby being associated with poorer diet quality and an increased risk of malnutrition [47, 48]. Inadequate nutritional intake and the accompanying deterioration in muscle function may further compromise the long-term maintenance of oral function [49].

Both previous studies and the present study indicate that female participants constitute a high-risk group for oral frailty [17], and this disparity is likely attributable to the cumulative effects of multiple factors. Females have a longer life expectancy and a higher proportion of individuals in the oldest age groups. And together with postmenopausal estrogen decline—which contributes to periodontal tissue degeneration and an increased risk of tooth loss—as well as the high prevalence of xerostomia during the perimenopausal period and its detrimental effects on oral function, these factors may collectively increase their risk [50–52]. Meanwhile, observed sex differences may also be confounded by factors such as socioeconomic status and oral healthcare utilization [53]. Thus, female sex should currently be regarded only as a signal of increased risk for oral frailty, and prospective studies are needed to clarify the specific contributions of biological and socio-behavioral factors.

Only two studies on GSEOH were included in this review. Although the pooled results suggested a potential association between higher GSEOH scores and the risk of oral frailty in older adults, this finding showed borderline statistical significance (*p* = 0.05, 95% CI: 0.92–1.00) and substantial heterogeneity (*I*² = 77%). These results are limited by the small number of studies, small sample sizes, and lack of standardized measurement methods. Therefore, these conclusions should be regarded as preliminary rather than definitive. Future large-sample prospective studies using standardized measurement protocols are needed to clarify its role, and in clinical practice, it may be considered a potential indicator of interest for oral frailty.

In addition to the factors eligible for meta-analysis, we also identified 35 additional determinants reported in single studies with consistent directions of evidence, which exhibited a multi-domain clustering pattern. These determinants included sociodemographic factors (e.g. lower income and social frailty), oral health status indicators (e.g. periodontal disease and difficulties in mastication or swallowing), health status and physical function factors (e.g. multimorbidity, polypharmacy, history of stroke, and slower gait speed), and nutritional and lifestyle factors (e.g. reduced dietary diversity, abnormal nutritional screening results, smoking, and insufficient physical activity). Overall, these findings suggest that the development of oral frailty results from the interplay of multiple determinants across oral, systemic, behavioral, and social domains.

From a clinical practical perspective, early identification and comprehensive management should be strengthened among high-risk older adults, particularly those of advanced age, those with physical frailty, those with fewer than 20 remaining teeth, and female individuals. Interventions could integrate routine assessment of oral function and dentition, nutritional screening and support, frailty management, and targeted oral health education, and be implemented through multidisciplinary collaboration among dental, geriatric, and nutrition services.

This meta-analysis has several limitations. First, substantial heterogeneity persisted in the pooled prevalence estimates. Although subgroup analyses and univariable meta-regression identified country, assessment instrument, and publication year as important contributors, the residual heterogeneity suggests that other unmeasured factors may be involved. Second, most included studies were cross-sectional, and the identified risk factors therefore primarily reflect associations rather than temporal ordering or causality. Moreover, the limited number of cohort studies indicates that additional longitudinal evidence is needed. Third, most studies were of moderate to high quality. Residual bias, particularly in moderate-quality and cross-sectional studies, may have influenced the pooled prevalence and risk factor estimates; therefore, these results should be interpreted with caution. Fourth, the evidence was largely derived from Japan and China, which may limit generalizability to other settings. Fifth, we restricted assessment instruments to OFI-6/OFI-8 and included only English-language studies. While this approach may enhance data comparability, it may also introduce selection bias and limit the generalizability of the findings. Univariable meta-regression still showed that the measurement instrument explained more than half of the between-study variance, suggesting that differences in instrument composition, thresholds, and implementation details may have introduced structural shifts. We note that differences in covariate adjustment across studies may contribute to heterogeneity and limit the interpretability of pooled associations. Residual confounding cannot be fully excluded, so these associations should be interpreted cautiously. Future research should conduct prospective cohort studies across multiple regions and settings under a standardized measurement framework, and undertake intervention studies targeting modifiable components to clarify preventable and controllable pathways of oral frailty and optimize management strategies.

## Conclusions

This systematic review and meta-analysis included 16 studies (*n* = 13,030) and showed that oral frailty is common among older adults, with a pooled prevalence of 32% (95% CI: 25%–41%). Subgroup analyses and univariable meta-regression indicated that country, assessment instrument, and publication year were significantly associated with prevalence estimates, with the assessment instrument explaining the greatest proportion of heterogeneity (R² = 56%), highlighting the need for caution in cross-study comparisons and underscoring the urgency of standardized assessment and cross-cultural validation. Meta-analyses of risk factors showed that older age, physical frailty, having fewer than 20 remaining teeth, and female sex were associated with an increased risk of oral frailty, whereas the evidence regarding GSEOH remains inconclusive. Early identification and comprehensive interventions for high-risk groups in community and healthcare settings are recommended, and future prospective longitudinal studies using unified definitions and standardized measurement tools are needed to validate risk prediction and intervention effects. However, as most included studies were conducted in Japan and China, caution is warranted when generalizing these prevalence estimates to other populations or regions.

## Supplementary Information

Supplementary Material 1.

Supplementary Material 2.

## Data Availability

Data is provided within the manuscript or supplementary information files.

## Abbreviations

OFI-6: Oral Frailty Index-6
OFI-8: Oral Frailty Index-8
GSEOH: Geriatric self-efficacy for oral health
NOS: Newcastle-Ottawa Scale
AHRQ: Agency for Healthcare Research and Quality
HbA1c: Glycated hemoglobin
MNA-SF: Mini Nutritional Assessment-Short Form
BMI: Body Mass Index
