# Supplementary material for: A systematic review and meta-analysis of the prevalence and risk factors of oral frailty among older adults
Source: BMC Oral Health. 2026 May 2;26:1168. doi: 10.1186/s12903-026-08435-y (PMC13325997; doi:10.1186/s12903-026-08435-y)
Supplement: Supplementary file 2 — Supplementary Material 2. [file 12903_2026_8435_MOESM2_ESM.docx]

**Supplementary Material**

**Supplementary Table 1.** Search strategy

| Database | No | Query | Results |
| --- | --- | --- | --- |
| PubMed | #1 | "Aged"[MeSH Terms] | 3729628 |
|  | #2 | "elderly"[Title/Abstract] OR "older adult*"[Title/Abstract] OR "older people"[Title/Abstract] OR "senior*"[Title/Abstract] OR "elder"[Title/Abstract] | 551346 |
|  | #3 | #1 OR #2 | 3913253 |
|  | #4 | "oral frailty"[Title/Abstract] | 215 |
|  | #5 | "impact*"[Title/Abstract] OR "cause*"[Title/Abstract] OR "reason*"[Title/Abstract] OR "association"[Title/Abstract] OR "relationship"[Title/Abstract] OR "effect*"[Title/Abstract] | 14569466 |
|  | #6 | #3 AND #4 AND #5 | 126 |
| Web of Science | #1 | TS=(aged or elderly or older adult* or older people or senior* or elder) | 4,831,535 |
|  | #2 | TS=(oral frailty) | 1,361 |
|  | #3 | TS=(impact* or cause* or reason* or association or relationship or effect*) | 21,298,938 |
|  | #4 | #1 AND #2 AND #3 | 939 |
| Embase | #1 | ('aged'/exp OR 'aged') AND [embase]/lim | 4,886,423 |
|  | #2 | ('aged patient':ti,ab,kw OR 'aged people':ti,ab,kw OR 'aged person':ti,ab,kw OR 'aged subject':ti,ab,kw OR 'elderly':ti,ab,kw OR 'elderly patient':ti,ab,kw OR 'elderly people':ti,ab,kw OR 'elderly person':ti,ab,kw OR 'elderly subject':ti,ab,kw OR 'senior citizen':ti,ab,kw OR 'senium':ti,ab,kw OR 'aged':ti,ab,kw) AND [embase]/lim | 1769532 |
|  | #3 | #1 OR #2 | 4,961,049 |
|  | #4 | "oral frailty"ti,ab,kw | 228 |
|  | #5 | impact*: ti,ab,kw OR cause*: ti,ab,kw OR reason*: ti,ab,kw OR association:ti,ab,kw OR relationship:ti,ab,kw OR effect*: ti,ab,kw | 19,017,924 |
|  | #6 | #3AND #4 AND #5 | 75 |
| Cochrane Library | #1 | MeSH descriptor: [Aged] in all MeSH products | 282778 |
|  | #2 | (aged or ciderty or older adult" or older poopie or senlor" or clderjtl,ab,kw | 1020142 |
|  | #3 | #1 OR #2 | 1020142 |
|  | #4 | (oral frailty): ti,ab,kw | 332 |
|  | #5 | (impact* or cause* or reason* or association or relationship or effect*): ti,ab,kw | 1562292 |
|  | #6 | #3 AND #4 AND #5 | 244 |
| CINAHL | S1 | MH aged OR TI (aged or elderly or older adult* or older people or senior* or elder) | 1,018,264 |
|  | S2 | AB aged or elderly or older adult* or older people or senior* or elder | 388,819 |
|  | S3 | S1 OR S2 | 1,207,253 |
|  | S4 | TI oral frailty OR AB oral frailty | 205 |
|  | S5 | TI (impact* or cause* or reason* or association or relationship or effect*) OR AB (impact* or cause* or reason* or association or relationship or effect*) | 2,648,152 |
|  | S6 | [S3 AND S4 AND S5](http://research--ebsco--com--https.ebscohost.shd1rmyy.lwnote.com:50001/search/results?combinedSearchQueryId=sq:0e86c3b3-4937-4c09-b867-266006746f31&db=ccm&expanders=concept&limiters=None&q=S3%20AND%20S4%20AND%20S5&searchMode=boolean&sort=relevance&userDirectAction=true&isDashboardExpanded=true) | 133 |
| MEDLINE | #1 | ('aged'/exp OR 'aged') AND [medline]/lim | 4,760,080 |
|  | #2 | (elderly:ab,ti OR 'older adult*':ab,ti OR 'older people':ab,ti OR senior*:ab,ti OR elder:ab,ti) AND [medline]/lim | 453,606 |
|  | #3 | #1 OR #2 | 4,869,025 |
|  | #4 | oral frailty': ab,ti AND [medline]/lim | 156 |
|  | #5 | (impact*: ab,ti OR cause*: ab,ti OR reason*: ab,ti OR association:ab,ti OR relationship:ab,ti OR effect*: ab,ti) AND [medline]/lim | 12,005,059 |
|  | #6 | #3 AND #4 AND #5 | 98 |

**Supplementary Table 2.** Excluded studies following full text screening with reasons

| First author year | Excluded articles | Reason |
| --- | --- | --- |
| Yu, J 2025 | Associated factors of oral frailty in older adults with long-term T2DM duration of more than 10 years | The outcome indicators do not match |
| Suzuki, F 2024 | Association between animal protein intake, oral frailty and calf circumference in middle-aged and older adults: a cross-sectional analysis from the Shika study | The outcome indicators do not match |
| Sun, Yuanhao 2025 | Association of social frailty, sarcopenia, and oral frailty with depressive symptoms in Chinese older adults: a cross-sectional study | The outcome indicators do not match |
| Masanori, Iwasaki 2024 | Prevalence of oral frailty and its association with dietary variety, social engagement, and physical frailty: Results from the Oral Frailty 5-Item Checklist | The outcome indicators do not match |
| Miku, Izutsu 2023 | Risk factors for oral frailty among community-dwelling pre-frail older adults in Japan: A cross-sectional analysis | The assessment tool does not match |
| Kobayashi, Yu 2025 | Association of Oral Frailty with Physical Frailty and Malnutrition in Patients on Peritoneal Dialysis | The study population does not match |
| Irie, K. 2024 | Relationship between risk of oral frailty and awareness of oral frailty among community-dwelling adults: a cross-sectional study | The study population does not match |
| Yu, J. 2024 | The association between oral frailty and HbA1c among older adults with T2DM: the chain mediating effect of nutritional status and physical frailty | The outcome indicators do not match |
| Yin, Z. H. 2020 | Eating and communication difficulties as mediators of the relationship between tooth loss and functional disability in middle-aged and older adults | The outcome indicators do not match |
| Hu, Xiao‐yi 2025 | Associations between oral frailty, oral microbiota composition, and postoperative delirium in older adult patients | The outcome indicators do not match |
| Ya, Su 2025 | ASSOCIATION BETWEEN ORAL FRAILTY AND INTRINSIC CAPACITY AMONG COMMUNITY-DWELLING OLDER ADULTS | abstract |
| Kusunoki, Hiroshi 2024 | Oral Frailty and Its Relationship with Physical Frailty in Older Adults: A Longitudinal Study Using the Oral Frailty Five-Item Checklist | The outcome indicators do not match |
| Baba, H. 2022 | Oral frailty and carriage of oral Candida in community-dwelling older adults (Check-up to discover Health with Energy for senior Residents in Iwamizawa; CHEER Iwamizawa) | The outcome indicators do not match |
| Iwasaki, M. 2020 | Association between Oral Frailty and Nutritional Status Among Community-Dwelling Older Adults: The Takashimadaira Study | The outcome indicators do not match |
| Iwasaki, M.2024 | Association between oral frailty and nutritional status among hemodialysis patients aged ≥50 years | The study population does not match |
| Iwasaki, M.2021 | Original Oral frailty and gait performance in community-dwelling older adults: findings from the Takashimadaira study | The outcome indicators do not match |
| Kamide, N.2024 | The association of oral frailty with fall risk in community-dwelling older adults: a cross-sectional, observational study | The outcome indicators do not match |
| Kimura 2024 | Association between oral frailty and Prevotella percentage in the oral microbiota of community‐dwelling older adults who participated in the CHEER Iwamizawa project, Japan | The outcome indicators do not match |
| Kugimiya, Y.2020 | Rate of oral frailty and oral hypofunction in rural community-dwelling older Japanese individuals | The outcome indicators do not match |
| Lin, Y. C.2022 | Physical Frailty and Oral Frailty Associated with Late-Life Depression in Community-Dwelling Older Adults | The assessment tool does not match |
| Song 2024 | The mediating effect of nutrition on oral frailty and fall risk in community-dwelling elderly people | The outcome indicators do not match |
| Tanaka 2018 | Oral Frailty as a Risk Factor for Physical Frailty and Mortality in Community-Dwelling Elderly | The outcome indicators do not match |
| Watanabe, D 2024 | Oral frailty is associated with mortality independently of physical and psychological frailty among older adults | The outcome indicators do not match |

**Supplementary Table 3.** Literature Quality Evaluation

A

Methodological quality appraisal results based on the AHRQ tool for each study.

| **Study** | **Item 1** | **Item 2** | **Item 3** | **Item 4** | **Item 5** | **Item 6** | **Item 7** | **Item 8** | **Item 9** | **Item 10** | **Item 11** | **Total score** | **Quality** |
| --- | --- | --- | --- | --- | --- | --- | --- | --- | --- | --- | --- | --- | --- |
| Kumar 2023 | Y | Y | Y | Y | U | Y | U | Y | U | U | U | 6 | M |
| Luo 2025 | Y | Y | Y | N | U | Y | Y | Y | Y | Y | U | 8 | H |
| Tian 2025 | Y | Y | Y | N | U | Y | U | Y | U | U | U | 5 | M |
| Hironaka 2020 | Y | Y | Y | N | U | Y | Y | Y | Y | Y | U | 8 | H |
| Komatsu 2021 | Y | Y | Y | N | U | Y | Y | Y | Y | U | U | 7 | M |
| Ohara 2020 | Y | Y | Y | N | U | Y | Y | Y | Y | Y | U | 8 | H |
| Hoshino 2021 | Y | Y | Y | N | U | Y | Y | Y | Y | Y | U | 8 | H |
| Nakagawa 2024 | Y | Y | Y | N | U | Y | Y | Y | Y | U | U | 7 | M |
| Yamamoto 2022 | Y | Y | Y | U | U | U | Y | Y | Y | U | U | 6 | M |
| Tamaki,N 2024 | Y | Y | Y | N | U | Y | Y | Y | Y | U | U | 7 | M |
| Liu 2025 | Y | Y | Y | U | U | Y | U | Y | N | U | U | 5 | M |
| Wang 2024 | Y | Y | Y | N | U | Y | U | Y | U | Y | U | 6 | M |
| Zhang 2025 | Y | Y | Y | N | U | Y | U | Y | U | U | U | 5 | M |
| Ishii 2022 | Y | Y | Y | U | U | U | Y | Y | Y | U | U | 6 | M |

Note: Y, yes; N, no; U, unclear; H, high quality; M, medium quality.

Item 1: Define the source of information (survey, record review).

Item 2: List inclusion and exclusion criteria for exposed and unexposed subjects (cases and controls) or refer to previous publications.

Item 3: Indicate time period used for identifying patients.

Item 4: Indicate whether or not subjects were consecutive if not population-based.

Item 5: Indicate if evaluators of subjective components of study were masked to other aspects of the status of the participants.

Item 6: Describe any assessments undertaken for quality assurance purposes (e.g., test/retest of primary outcome measurements).

Item 7: Explain any patient exclusions from analysis.

Item 8: Describe how confounding was assessed and/or controlled.

Item 9: If applicable, explain how missing data were handled in the analysis.

Item 10: Summarize patient response rates and completeness of data collection.

Item 11: Clarify what follow-up, if any, was expected and the percentage of patients for which incomplete data or follow-up was obtained.

B

Methodological quality appraisal results based on the NOS tool for each study.

| **Study** | **Selection** |  |  |  | **Comparability** | **Outcome** |  |  | **Total** | **Quality** |
| --- | --- | --- | --- | --- | --- | --- | --- | --- | --- | --- |
|  | **Item 1** | **Item 2** | **Item 3** | **Item 4** | **Item 5** | **Item 6** | **Item 7** | **Item 8** |  |  |
| Nishimoto 2023 | ★ | ★ | ★ | ★ | ★★ | ★ | ★ | ND | 8 | H |
| Iwasaki 2020 | ★ | ★ | ★ | ★ | ★★ | ★ | ★ | ND | 8 | H |

Note: H, high quality; M, medium quality; ND, not described

Item 1: Representativeness of the exposed cohort.

Item 2: Selection of the non exposed cohort.

Item 3: Ascertainment of exposure.

Item 4: Demonstration that outcome of interest was not present at start of study.

Item 5: Comparability of cohorts on the basis of the design or analysis.

Item 6: Assessment of outcome.

Item 7: Was follow-up long enough for outcomes to occur.

Item 8: Adequacy of follow up of cohorts.

**Supplementary Figure 1.** Sensitivity analysis of prevalence of oral frailty

**Supplementary Figure 2.** Sensitivity analysis of risk factors for oral frailty


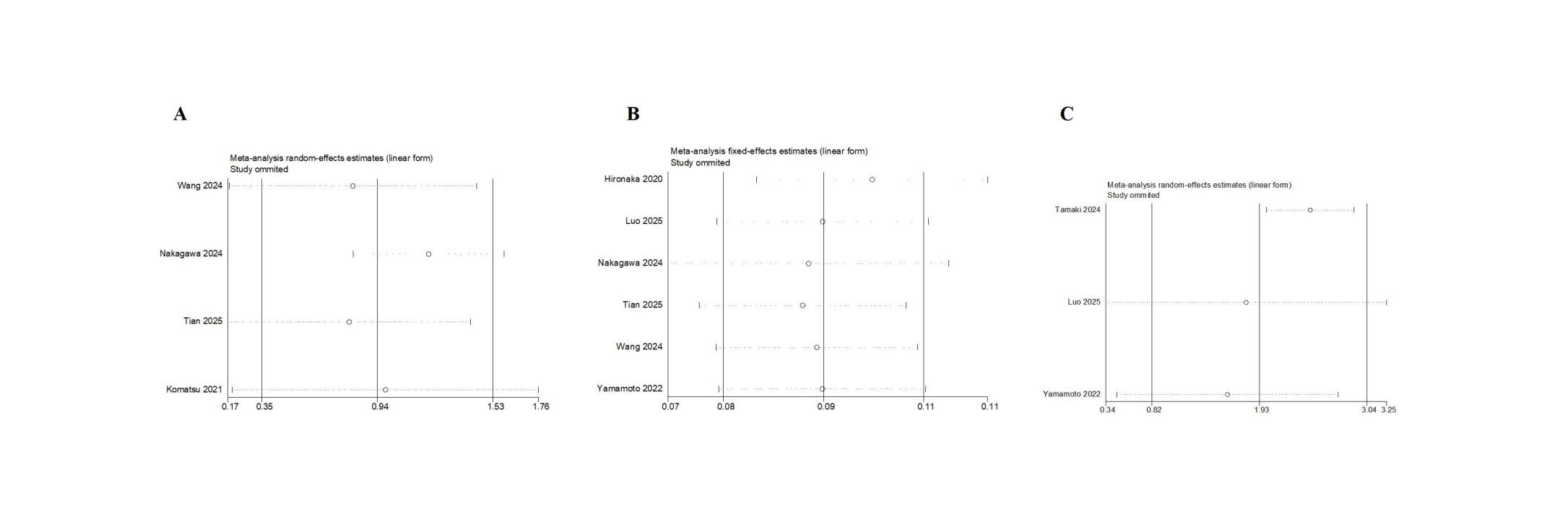


(A) Physical frailty ,(B)Age,(C) the number of remaining teeth.

**Supplementary Figure 3.** Assessment of the prevalence of oral frailty with publication bias funnel plot

**Supplementary Table 4.** Subgroup analysis of the prevalence of oral frailty

| Subgroup | Number of included report | Oral frailty | |  |  |  | Difference between groups | |
| --- | --- | --- | --- | --- | --- | --- | --- | --- |
|  |  | Prevalence | 95%CI | P value | Effect model | I^2^(%) | Qbet | P value |
| Country | | | | | | | 8.709 | 0.003 |
| China | 5 | 48.7 | 0.352-0.623 | ＜0.001 | Random | 97.49 |  |  |
| Japan | 10 | 24.9 | 0.173-0.334 | ＜0.001 | Random | 97.83 |  |  |
| Mean age | | | | | | | 1.30 | 0.254 |
| ＜74 | 4 | 19.2 | 0.106-0.295 | ＜0.001 | Random | 97.359 |  |  |
| ≥74 | 5 | 29.2 | 0.157-0.449 | ＜0.001 | Random | 98.89 |  |  |
| Study design | | | | | | | 10.994 | 0.001 |
| Cross-sectional | 13 | 34.7 | 0.263-0.437 | ＜0.001 | Random | 98.831 |  |  |
| Cohort study | 2 | 20.5 | 0.187-0.225 | ＜0.001 | Random | 98.851 |  |  |
| Measurement | | | | | | | 13.757 | <0.001 |
| OFI-6 | 8 | 20.6 | 0.121-0.306 | ＜0.001 | Random | 98.9 |  |  |
| OFI-8 | 7 | 47.6 | 0.376-0.578 | ＜0.001 | Random | 97.572 |  |  |
| Publishing year | | | | | | | 16.65 | <0.001 |
| Before2022 | 7 | 20.6 | 0.146-0.274 | ＜0.001 | Random | 95.391 |  |  |
| 2023-2025 | 8 | 43.3 | 0.348-0.520 | ＜0.001 | Random | 98.424 |  |  |
| Samp size |  |  |  |  |  |  | 0.485 | 0.486 |
| ≤500 | 8 | 35.5 | 0.21-0.515 | ＜0.001 | Random | 98.739 |  |  |
| ＞500 | 7 | 29.1 | 0.197-0.395 | ＜0.001 | Random | 99.096 |  |  |
| Study setting | | | | | | | 2.485 | 0.115 |
| Community | 9 | 27.9 | 0.174-0.398 | ＜0.001 | Random | 99.172 |  |  |
| Hospital/clinic | 6 | 39.4 | 0.313-0.478 | ＜0.001 | Random | 95.678 |  |  |

**Supplementary Table 5.** Meta-regression of prevalence of oral frailty

| Factors | OR | 95%CI | Adjust R^2^(%) | P value |
| --- | --- | --- | --- | --- |
| Japan | 0.80 | 0.68 – 0.93 | 36.0 | 0.004 |
| Cohort study | 0.84 | 0.65 – 1.09 | 4.6 | 0.195 |
| OFI-6 | 0.77 | 0.68 – 0.87 | 56.1 | <0.001 |
| Publishing year |  |  |  |  |
| 2023-2025 | 1.24 | 1.08 – 1.43 | 37.2 | 0.003 |
| Sample size |  |  |  |  |
| ≤500 | 1.07 | 0.89 – 1.28 | 0.0 | 0.496 |
| Medical setting |  |  |  |  |
| Hospital/clinic | 1.11 | 0.93 – 1.33 | 2.0 | 0.255 |
